# The Role of Interfascial Plane Blocks in Paediatric Regional Anaesthesia: A Narrative Review of Current Perspectives and Updates

**DOI:** 10.1155/2020/8892537

**Published:** 2020-12-19

**Authors:** Sujana Dontukurthy, Roshanak Mofidi

**Affiliations:** ^1^Nationwide Children's Hospital, 700 Children's Drive, Columbus, OH 43035, USA; ^2^Keck Hospital of USC, 1520 San Pablo Street, Los Angeles, CA 90033, USA

## Abstract

Regional anaesthesia has been increasingly used for analgesia in the perioperative period in paediatric anaesthesia for better pain control and improved patient outcomes. Interfascial plane blocks are considered as a subgroup of peripheral nerve blocks. The advent of ultrasound in modern regional anaesthesia practice has led to the evolution of various interfascial plane blocks. The ease of their performance and the low complication rates, compared with neuraxial anaesthesia, have led to their increased use in the perioperative period. Interfascial plane blocks are often incorporated in the multimodal analgesia regimen in the early recovery and ambulation after surgery protocols for various chest wall and abdominal surgeries. This achieves better pain control and decreases the requirements of opioids in the perioperative period, thereby facilitating early mobilization and discharge. This narrative review focuses on the relevant anatomic considerations, technique for the performance of each block along with its current applications and limitations, and includes a review of the current literature on various interfascial plane blocks in paediatric regional anaesthesia.

## 1. Introduction

Interfascial plane blocks can significantly contribute to intraoperative and postoperative analgesia. When used for the appropriate surgical procedure and incorporated into multimodal analgesia regimens, interfascial blocks can provide good postoperative analgesia and decrease opioid requirements [1].

The advent of ultrasound has led to the evolution of various interfascial plane blocks for better pain management of chest and abdominal wall surgeries and has also significantly improved the scope of pain management in paediatric anesthesia [2, 3]. Ultrasound has further increased the success rate of these blocks, decreased the dosage requirements of the local anaesthetics compared to the traditional landmark technique, and allowed the visualization of the spread of local anaesthetic along the desired fascial planes to anesthetize the target nerves [3].

Unlike neuraxial anaesthesia, interfascial plane blocks are generally considered to have a safer adverse effect profile [4]. These simple blocks can also be used in thoracic and abdominal surgeries where neuraxial anaesthesia is not contraindicated [3]. Further, with the implementation of enhanced recovery after surgery (ERAS) protocols and given the concerns about the ongoing opioid epidemic, decreasing opioid use is critical. Decreasing the usage of opioids and better pain control can also help with early ambulation and a shorter length of hospital stay [3].

This review focuses on the anatomic considerations of the various chest wall and truncal interfascial plane blocks, the technique of administration and their current indications and limitations.

## 2. Blocks of the Chest Wall

### 2.1. Pecs Block

The pectoral muscles are supplied by the lateral and medial pectoral nerves. The lateral pectoral nerve arises from the roots of the T5-T7 cervical nerves and the lateral cord of the brachial plexus. It traverses the deeper surface of the pectoralis major muscle and provides sensory supply to the shoulder and the periosteum of the clavicle [5]. The medial pectoral nerve arises from the roots of the C8-T1 nerves and the medial cord of the brachial plexus and runs along the deeper surface of the pectoralis minor muscle. It provides innervation to the posterior and lateral aspects of the pectoralis minor and the axillary region of the chest wall [5].

**Figure 1:**
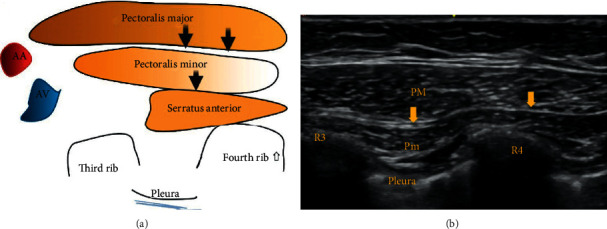
AA: axillary artery; AV: axillary vein. R3: third rib; R4: fourth rib. Black and yellow arrows indicate the point of local anesthetic infiltration.

There are two types of Pecs blocks. The Pecs I block is an interfascial plane block, performed with the transducer probe perpendicular to the rib cage and below the clavicle. The local anaesthetic is injected between the pectoralis major and pectoralis minor muscles, to block the lateral and medial pectoral nerves [6] (Figure 1).

The Pecs II block is a modification of the Pec I block. It involves the infiltration of local anaesthetic agents beneath the pectoralis minor muscle at the level of the third rib in the axilla to cover the pectoral compartment and the intercostal branches [7], and the Pecs II block has a widespread coverage and anesthetizes the pectoral, intercostobrachial, third to sixth intercostal, long thoracic, and thoracodorsal nerves. It has been shown to provide effective analgesia similar to paravertebral block [4].

### 2.2. Serratus Anterior Plane Block

**Figure 2:**
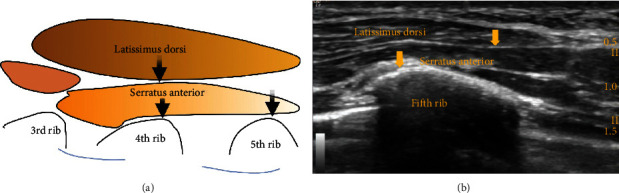
Black and yellow arrows indicate the point of local anesthetic infiltration.

The serratus plane block is performed at the level of the fourth and fifth rib spaces, more lateral and posterior to the Pecs I and II blocks in the axillary region. The local anaesthetic is infiltrated between the serratus anterior and latissimus dorsi muscles and beneath the serratus anterior muscle to anesthetize the intercostobrachial nerve, lateral cutaneous branches of the intercostal nerves (T3-T9), long thoracic nerve, and thoracodorsal nerve [8] (Figure 2).

### 2.3. Clinical Indications of Chest Wall Blocks

Even though the literature on chest wall blocks in paediatrics is sparse compared to that in adults, studies have shown the successful use of these blocks in surgeries such as thoracotomy, implantation of pacemakers and defibrillators, and in minimally invasive pectus excavatum repairs, where they reduced the opioid requirements and resulted in better pain scores in the postoperative period. Kaushal and colleagues [9] performed a randomized trial in paediatric patients with congenital heart disease who underwent cardiac surgery via thoracotomy, without bypass, and concluded that although the pain scores in the immediate postoperative period were similar between the three groups, the serratus anterior block and Pecs II fascial blocks provided longer-lasting analgesia than intercostal nerve blocks. However, the pain scores of Pecs II blocks were similar to intercostal nerve blocks at 12 hours. Rescue opioid consumption was significantly higher in the intercostal nerve block group. In a retrospective study by Altun and colleagues [10] in patients who underwent minimally invasive repair of pectus excavatum, bilateral serratus anterior blocks reduced the requirements for postoperative opioids and yielded lower pain scores as compared to patients who received only patient-controlled analgesia. Similar results were found with Pecs I and II blocks in a retrospective study conducted by Yang and colleagues [11] in paediatric patients undergoing pacemaker or defibrillator implantations.

### 2.4. Truncal Blocks

Truncal nerve blocks are technically simple to perform and, in combination with other analgesic techniques, can provide an analgesic efficacy that is similar to neuraxial analgesia [12]. In contrast to the traditional landmark techniques, ultrasound-guided techniques have increased the success rates of these blocks and decreased the dosages of local anaesthetics used.

### 2.5. Rectus Sheath Block

The rectus abdominis is an anterior abdominal wall muscle, divided in the midline by the linea alba. The rectus abdominis muscle is wide and thin superiorly and increases in thickness inferiorly. Most of the muscle is enclosed by the rectus sheath [13]. The thoracolumbar nerves (T9-T11) lie posterior to muscle and anterior to the posterior rectus sheath [3].

**Figure 3:**
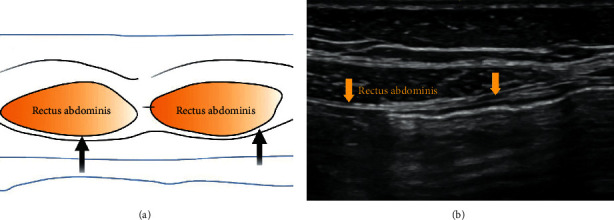
Black and yellow arrows indicate the point of local anesthetic infiltration.

The ultrasound transducer probe is placed in the transverse plane lateral to the umbilicus (Figure 3). The local anaesthetic solution is injected into the potential space between the posterior rectus sheath and the rectus abdominis muscle [13].

### 2.6. Clinical Indications of the Rectus Sheath Block

The rectus sheath block is frequently utilized to provide postoperative pain relief for surgeries involving midline incisions, particularly those utilizing vertical incisions, such as umbilical hernia repair [14] and single-incision laparoscopic surgery [13], in paediatric patients. The rectus sheath block has been shown to be effective for pain relief in the perioperative period in paediatric patients who have undergone umbilical hernia repair, but the depth of the posterior rectus sheath cannot be predicted based on age, height, and weight; thus, ultrasound should be utilized to perform a successful rectus sheath block [15]. This block has also been shown to provide superior analgesia in paediatric patients requiring midline incisions when undergoing laparoscopic surgery, in the immediate postoperative period as compared to the infiltration of local anaesthetics. There was no difference noted in the pain scores at 60 minutes between the two groups [13]. The spread of the local anaesthetic may be limited when using a single injection, and multiple injections may be required to target the desired dermatomes [16].

Rectus sheath block performed under ultrasound guidance has a good safety profile with minimal complications. However, advancing the needle too far can result in inadvertent puncture of the abdominal organs [3].

### 2.7. Transversus Abdominis Plane (TAP) Block

TAP blocks are often used as a part of multimodal analgesic regimens for anterior abdominal surgeries. The three muscles of the anterior abdominal wall—external oblique, internal oblique, and transversus abdominis muscles—help to support the abdominal contents and trunk. Beneath these muscles lie the extraperitoneal fat and parietal peritoneum. The rectus abdominis is the vertical midline abdominal muscle. The anterior rami of the T7-L1 thoracolumbar nerves provide sensory innervation of the skin, muscles, and parietal peritoneum and lie in the plane between the internal oblique and transversus abdominis muscles. The T7-T9 nerves supply the skin above the umbilicus, the T10 nerve supplies the umbilicus, while the skin inferior to the umbilicus is innervated by the T11 nerve, cutaneous branch of the subcostal T12 nerve, iliohypogastric nerve, and ilioinguinal nerve originating from L1.4.

**Figure 4:**
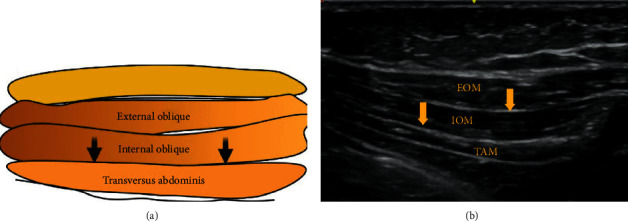
EOM: external oblique muscle; IOM: internal oblique muscle; TAM: transversus abdominis muscle. Black and yellow arrows indicate the point of local anesthetic infiltration.

To deliver a TAP block, the local anaesthetic is infiltrated between the internal oblique and transversus abdominis muscles, with the transducer probe placed in the anterior axillary line between the costal margin and the iliac crest. The posterior TAP block is administered by injecting the local anaesthetic posterior to the termination of the transversus abdominis muscle where the thoracolumbar fascia overrides the quadratus lumborum muscle [17]. For the procedure above the umbilicus, a subcostal technique provides better analgesia (Figure 4).

### 2.8. Clinical Indications of TAP Blocks

TAP blocks are used to provide analgesia for surgery of the anterolateral abdominal wall. It provides only somatic analgesia. In a randomized trial conducted by Bryskin et al. [18] in 45 children who underwent bilateral ureteral reimplantation, TAP blocks provided superior analgesia as compared with caudal blocks at 6–24 h after block placement but appeared to be less effective than caudal blocks in preventing viscerally mediated bladder spasms. In a study of children who underwent open appendectomy, administration of unilateral TAP blocks resulted in a significant reduction of morphine consumption for the first 48 hours after surgery [19]. Preoperative administration of TAP blocks for laparoscopic surgeries resulted in greater beneficial effects on early pain and opioid consumption than when administered in the postoperative period; larger doses of the local anaesthetic also had a superior effect on late postoperative pain at rest and on opioid consumption [20]. A study in children undergoing lower abdominal surgery showed similar results, with higher local anaesthetic doses having a prolonged analgesic duration (bupivacaine 2.5 mg/kg) than lower doses (bupivacaine 1.25 mg/kg) [21]. However, no differences in pain scores were found based on the volume of the local anaesthetic used [22].

The TAP block has a good safety profile; nevertheless, a few reports in adult literature have described liver damage and bowel hematoma [23, 24].

### 2.9. Quadratus Lumborum Block (QL)

The quadratus lumborum (QL) block is a relatively novel, deep fascial plane block that has been successfully used in various abdominal surgeries.

The quadratus lumborum (QL) muscle lies in the posterior abdominal wall, dorsolateral to the psoas major muscle. The thoracolumbar fascia (TLF) is a sheet of fused aponeuroses and fascial layers that covers the muscles of the back. The TLF is divided into anterior, middle, and posterior layers. It plays an important role to serve the spread of infiltrated local anaesthetics into the thoracic paravertebral space and contains a high-density network of sympathetic fibres and mechanoreceptors that are responsible for the effects of the block [25].

### 2.10. Types of QL Blocks and Techniques

Various types of QL blocks have been described in the literature. The most commonly performed are lateral, anterior, and posterior QL blocks.

To deliver the lateral QL block, the ultrasound probe is placed in the axial plane in the midaxillary line and moved posteriorly until the posterior aponeurosis of the transversus abdominis muscle becomes visible and the local anaesthetic is infiltrated deep to the posterior aponeurosis of the transversus abdominis muscle, but superficial to the thoracolumbar fascia, at the lateral margin of the QL muscle. It anaesthetizes the lateral cutaneous branches of the iliohypogastric, ilioinguinal, and subcostal nerves (T12-L1).

For the anterior QL block, the ultrasound probe is placed in the axial plane in the midaxillary line and moved posteriorly, until the QL muscle is visualized and the local anaesthetic is infiltrated between the psoas major and quadratus lumborum muscles.

**Figure 5:**
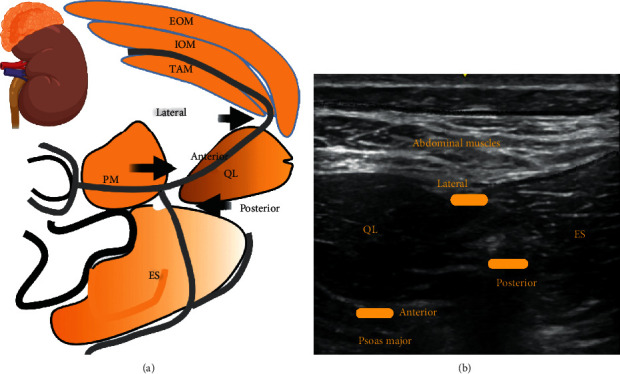
EOM: external oblique muscle; IOM: internal oblique muscle; TAM: transversus abdominis muscle; PM: psoas major; QL: quadratus lumborum, ES: erector spinae muscle. Black arrows indicate the point of local anaesthetic infiltration. Anterior, lateral, and posterior indicate the types of QL blocks and point of local anaesthetic infiltration. The grey lines represent thoracolumbar fascia.

For the posterior QL block, the point of injection is behind the QL muscle [24] (Figure 5).

### 2.11. Spread of Local Anaesthetic in the QL Blocks

Several cadaveric studies were performed to evaluate the spread of the dye in various types of QL blocks.

Carline and colleagues [26] evaluated the spread of dye and nerve involvement after performing four anterior, three lateral, and three posterior QL blocks using an ultrasound-guided technique with 20 ml of dye solution for each QL block. The anterior QL block consistently dyed the lumbar nerve roots and sometimes dyed nerves within the TAP. The posterior and lateral QL blocks almost reached the TAP and dyed the subcutaneous tissue surrounding the abdominal flank and the deep muscles of the back. A similar cadaveric study conducted by Elsharkawy and coworkers [27] to evaluate the spread of dye in the anterior and posterior QL blocks found that the anterior QL block has a more consistent mid-to-lower thoracic spread, while the posterior block has more spread of the injectate to the lateral and posterior abdominal wall, with a limited cephalad spread.

A prospective randomized trial conducted in canine cadavers to study the spread of the injectate using high (0.3 ml/kg) and low volumes (0.15 ml/kg) of the dye-lidocaine solution in the anterior QL block found that high volume solutions have more cephalad spread and stained the lumbar sympathetic trunk more compared to low volume solutions [28].

### 2.12. Clinical Indications of QL Blocks

One of the limitations of fascial plane blocks in general is their inability to provide visceral analgesia. However, the QL block provides somatic as well as visceral analgesia, making the QL block an effective analgesic tool for abdominal surgery. Currently, the QL block is performed for perioperative pain management procedures for individuals of all ages undergoing abdominal surgery.

In a study by Sato et al. [29] in paediatric patients undergoing bilateral ureteral implantation via lower abdominal incision for vesicoureteral reflux, the QL block was shown to be as effective as caudal ropivacaine blocks in the immediate postoperative period and had superior and longer analgesic effects at 24 hours postoperatively. A similar study in paediatric patients undergoing inguinal hernia repair with orchidopexy found that QL blocks provided more effective analgesia than caudal blocks without adjuvants [30]. A comparative study by Öksüz and colleagues [31] in 50 paediatric patients undergoing elective unilateral inguinal hernia repair or orchiopexy found that QL blocks provided superior and longer postoperative analgesia than TAP blocks. A comparison of QL blocks and ilioinguinal/iliohypogastric blocks in paediatric patients undergoing open inguinal herniorrhaphy by Samerchua et al. found that QL blocks provided superior pain control [32]. In a pilot study by Aksu and colleagues [33], 10 patients who received anterior QL blocks for inguinal hernia were pain-free for 48 hours after the surgery. In a similar pilot study by Baidya and coworkers [34] in five paediatric patients undergoing pyeloplasty through a lumbotomy approach, administration of the QL block via a posterior transmuscular approach resulted in a median time to the first dose of morphine of 5 hours and good postoperative analgesia. Visiou and colleagues [35] presented the case of a 5-year-old patient undergoing colostomy closure under general anaesthesia; continuous QL block was provided for 3 days using a catheter approach, which provided analgesia without any restriction of activity. The QL block is a deep interfascial plane block. The lower pole of the kidney lies anterior to the QL muscle and can reach the L4 level during deep inspiration. Caution should be exercised when performing the QL block to avoid kidney injury.

### 2.13. Erector Spinae Block

The erector spinae is a group of muscles that include the iliocostalis, longissimus, and spinalis muscles. These muscles run from the skull to the pelvis and sacral region and from the spinous to the transverse processes, extending to the ribs. This muscle group is encased in a retinaculum that extends from the skull to the sacrum.

**Figure 6:**
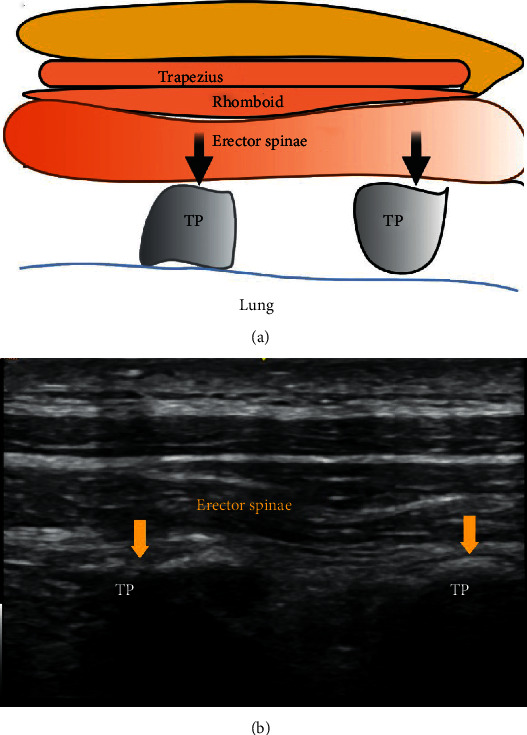
TP: transverse process. Black and yellow arrows indicate the point of local anaesthetic infiltration.

Ultrasound-guided erector spinae plane block (ESPB) is a paravertebral regional anaesthesia technique in which the local anaesthetic is deposited in the plane deep between the erector spinae muscles and the tips of the transverse processes of the vertebrae, with the ultrasound probe placed parallel to the vertebra. The retinaculum enclosing the muscle group provides an anatomical basis for the craniocaudal spread of the local anaesthetic (Figure 6).

The mechanism of analgesia in ESPB is complex, and various studies have shown different results. In a cadaveric study by Ivanusic and colleagues [36], extensive craniocephalad and lateral spread of the dye was observed and showed involvement of the dorsal ramus posterior to the region of the costotransverse foramen. Similar results were found in porcine models [37]. In contrast, Govender and colleagues [38] conducted a neonatal cadaveric study by injecting methylene blue for ESPB and concluded that the dye not only showed craniocaudal spread but was also found anteriorly in the paravertebral and epidural spaces, staining both the dorsal and ventral rami of the T2-T12 spinal nerves.

### 2.14. Clinical Indications of ESPB

ESPB has been used as a regional anaesthetic modality for thoracic and abdominal surgeries. It has also been successfully used for pain management in rib fractures. Even though it is a relatively new block, the ease of application and relative safety of the technique has garnered attention from clinicians.

ESPB at T12 has been used successfully for postoperative analgesia after paediatric nephrectomy and has been shown to be effective for 48 hours after surgery [39]. A similar experience was reported after ESPB at the T5 level in a 7-year-old patient after excision of a rib tumour, where it provided effective analgesia for up to 32 hours after surgery [40]. It has also been shown to be effective in patients undergoing pectus excavatum repair [41]. ESPB was shown to provide better pain control than thoracic epidural blocks after video-assisted thoracoscopic surgery [42]. In a case report by Paladini and colleagues [43], ESPB provided good intraoperative and postoperative analgesia for a patient who underwent thoracoscopic surgery for congenital pulmonary airway malformation. In a randomized control trial in 60 paediatric patients who underwent splenectomy, patients who received the ESPB had reduced opioid and nonopioid analgesic requirements for the first 8 hours after surgery in addition to reduced intraoperative opioid requirements, compared to the control group [44]. A retrospective randomized trial conducted by Holland and coworkers [45], in 164 children who underwent various thoracic and abdominal surgeries, including cardiac surgery, found that the ESPB provided good postoperative analgesia in various surgeries involving incisions from T1 to as low as L4, without any documented complications.

The ESPB is an interfascial plane block; therefore, the success of the block depends on the volume of local anaesthetic injected. Long-acting local anaesthetics or continuous infusions through catheters have typically been used.

### 2.15. Ilioinguinal/Iliohypogastric Nerve Block (IL/IH)

The ilioinguinal/iliohypogastric nerve block is indicated for inguinal hernia, orchidopexy, and hydrocoelectomy [46].

**Figure 7:**
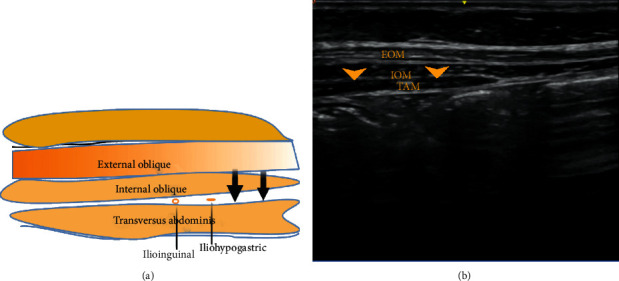
EOM: external oblique muscle; IOM: internal oblique muscle; TAM: transversus abdominis muscle. Black and yellow arrows indicate the point of local anaesthetic infiltration. The yellow dots indicate ilioinguinal and iliohypogastric nerves.

The ilioinguinal nerve and iliohypogastric nerves arise from the L1 nerve and emerge from the upper part of the lateral border of the psoas major muscle [3]. The ilioinguinal and iliohypogastric nerves innervate the posterolateral gluteal region, inguinal region, and anterior scrotum; they run just medial to the anterior superior iliac spine and between the internal oblique and transversus abdominis muscles. The block is performed with the ultrasound probe placed medial to the anterior superior iliac spine at the level of the umbilicus; the local anaesthetic is infiltrated between the internal oblique and transversus abdominis muscles [1] (Figure 7). However, the location of these nerves has been shown to vary significantly with age in paediatric patients. In a study performed by Hong et al. [47] in paediatric patients (1–82 months of age), the ultrasound evaluation of the point of needle insertion in an imaginary line drawn between the anterior superior iliac spine (ASIS) and the umbilicus showed that there is a considerable variation in the distance between the ASIS iliohypogastric nerve, ASIS ilioinguinal nerve, and in between the two nerves, among the different age groups. Therefore, age must be considered when locating these nerves while using the landmark technique.

### 2.16. Clinical Indications of Ilioinguinal/Iliohypogastric Nerve Block

Lee and colleagues [48] reported the successful use of ultrasound-guided ilioinguinal/iliohypogastric nerve blocks in six premature babies who underwent inguinal hernia repair under general anaesthesia. None of the patients required opioids in the perioperative period, and the volume of bupivacaine needed for the block was only 0.17 ml/kg, as compared to 0.25 ml/kg in the landmark-based technique. In a retrospective study performed by Thong and colleagues, [49] in 80 expremature neonates undergoing inguinal hernia repair under general anaesthesia with ilioinguinal/iliohypogastric nerve blocks, it was found that these blocks were effective in about 80% of the patients, as they did not require administration of rescue opioids postoperatively.

Weintraud and colleagues [50] conducted a prospective study in 62 children who received ilioinguinal/iliohypogastric nerve blocks based on the landmark technique and examined the actual location of the local anaesthetic using ultrasound. They concluded that, in the majority of patients, the local anaesthetic was inaccurately delivered to the adjacent structures, and it was correctly deposited around the nerves in only 14% of the cases. Therefore, the landmark-based technique can be unreliable for a successful ilioinguinal/iliohypogastric nerve block.

Overall, with the use of ultrasound, the ilioinguinal/iliohypogastric nerve block is relatively safe. Complications include infection, intravascular injection, local anaesthetic toxicity, bowel puncture, pelvic hematoma, and femoral nerve palsy [51]. Transient femoral nerve palsy with quadriceps weakness and numbness over the anterior aspect of the thigh has been reported after administration of large volumes of the local anaesthetic solution for block [52].

The dermatomal distribution covered by the block and the surgeries in which they are commonly used are presented in a tabular form in Table 1.

### 2.17. Cautions for Use of Fascial Plane Blocks in Children

Caution regarding local anaesthetic toxicity should be exercised in paediatric patients. The incidence of such toxicity may be higher than in adults, as the targeted tissue planes are very vascular, and the blocks require a high volume of local anaesthetic agent to ensure adequate spread in the fascial planes [53]. The potential for high plasma concentration is further increased by the higher cardiac output and local blood flow in children than in adults. Specific recommendations for these techniques include strict attention to the dosing guidelines (with dosing based on lean body weight), use of dilute local anaesthetic solutions to achieve the required volumes, addition of epinephrine to limit systemic absorption, use of less cardiotoxic local anaesthetic agents, and monitoring of the patient for 30–45 minutes after the block to allow achievement of peak plasma concentrations [53].  Table 2 provides the maximum suggested dosage of the local anesthetics in pediatrics.

## 3. Conclusion

With the introduction of ultrasound in regional anaesthesia practice, interfascial plane blocks have evolved in recent years and, in combination with other analgesics, can be used as the main component of multimodal postoperative analgesia. When performed by a skilled anaesthesiologist with appropriate caution, these blocks can provide effective pain relief in paediatric patients undergoing surgery with fewer complications. The recent literature supports the use of interfascial blocks in paediatric surgery as a component of multimodal analgesia. Further research is needed to evaluate the efficacy and safety of these blocks in paediatric regional anaesthesia.

## Figures and Tables

**Table 1:** The table shows various surgeries in which interfascial plane blocks are commonly used and the nerves targeted for the corresponding blocks.

Type of block	Targeted nerves	Surgery/indication
PEC blocks and serratus anterior block	Medial (C8-T1) and lateral pectoral (C5-C7), long thoracic nerve (C5-C7)	Thoracotomy and thoracoscopic procedures, breast surgeries, insertion cardiac resynchronization device, nuss procedures, and traumatic rib fractures
Rectus sheath block	T7-T12	For surgeries with midline abdominal incision
Transversus abdominis block	Lower six thoracic (T7-T12) and first lumbar nerve (L1)	Laparotomy, laparoscopic surgeries of the abdominal wall Provides only somatic analgesia
Ilioinguinal/iliohypogastric block	Iliohypogastric and ilioinguinal nerves arising from L1	Inguinal hernia repair, orchiopexy, and hydrocele repair
Quadratus lumborum block	Lateral QLB-L1 Anterior QLB-T4-L1 Posterior QLB-T4-L1	Lateral QLB provides analgesia for abdominal surgeries below the umbilicus Anterior and posterior QLB provides both somatic and visceral analgesia for abdominal surgeries above and below the umbilicus
Erector spinae block	Spread to anterior and posterior rami in craniocaudal direction depending on the site of injection	Found to be effective in various thoracic, breast, and abdominal surgeries

**Table 2:** Maximum recommended dosages of commonly used local anaesthetics in paediatric patients.

Local anaesthetic	Class	Maximum dose
Lidocaine	Amide	5 mg/kg
Bupivacaine	Amide	2.5 mg/kg
Ropivacaine	Amide	2.5 mg/kg
Levobupivacaine	Amide	2.5 mg/kg
2 Chloroprocaine	Ester	20 mg/kg

Adapted from NYSORA: Regional Anesthesia in Pediatric Patients: General Considerations.

## Data Availability

Data used in table are adapted from NYSORA.
